# Factors Influencing Exclusive Breastfeeding in Saudi Arabia

**DOI:** 10.3390/healthcare12060639

**Published:** 2024-03-12

**Authors:** Nawal Alissa, Mawaddah Alshareef

**Affiliations:** 1 College of Sport Sciences and Physical Activity, King Saud University, Riyadh 11362, Saudi Arabia; 2 Department of Community Health Sciences, College of Applied Medical Sciences, King Saud University, Riyadh 11362, Saudi Arabia; 3 Makkah Maternity and Childhood Hospital, Mecca 24246, Saudi Arabia; mawaddahalshreef@gmail.com

**Keywords:** exclusive breastfeeding, factors, Saudi Arabia

## Abstract

Background: Exclusive breastfeeding is defined as the practice of providing infants with breast milk as their sole source of nourishment for the first six months of life. This study investigated the factors influencing exclusive breastfeeding practices in Makkah, Saudi Arabia. Methods: The study employed a descriptive cross-sectional study design. Data gathered from 340 mothers attending the Maternity and Childhood Hospital in Makkah provided insights into the demographic profiles and postpartum practices of participants. Results: The study revealed the significance of early breastfeeding initiation and the provision of pre-birth breastfeeding information in extending the duration of exclusive breastfeeding. There was a statistically significant difference between mothers who had Cesarean section deliveries and those who had natural deliveries in terms of exclusive breastfeeding duration. Conclusions: These findings have essential implications for healthcare professionals, policymakers, and future research endeavors, emphasizing the importance of healthcare education and timely support in promoting extended exclusive breastfeeding practices.

## 1. Introduction

The promotion of exclusive breastfeeding, defined by the World Health Organization (WHO) as the practice of feeding an infant with breast milk alone for the first six months of life without any additional food or liquids, is an essential component of public health and infant well-being [1]. The importance of this practice is emphasized by the wealth of scientific evidence supporting its role in safeguarding infant and maternal health, as well as its broader societal implications.

Globally, there has been an increasing emphasis on improving exclusive breastfeeding rates, recognizing its potential to reduce infant morbidity and mortality, enhance cognitive development, and foster a secure maternal–child bond [2,3,4,5,6,7]. Although there are strong reasons supporting exclusive breastfeeding, the actual practice of exclusive breastfeeding remains negligible across the globe. Understanding the determinants of exclusive breastfeeding is crucial, not only for improving individual health outcomes but also for achieving broader public health goals [8,9,10,11].

### 1.1. Exclusive Breastfeeding in Saudi Arabia

Saudi Arabia, as a signatory to international health standards, adopts WHO recommendations on exclusive breastfeeding for the first six months of an infant’s life followed by the introduction of complementary foods while continuing to breastfeed for up to two years or beyond [12]. However, despite these guidelines and extensive national and international efforts, the rates of exclusive breastfeeding in Saudi Arabia still need to catch up to the recommended global targets.

In a study conducted on the state of exclusive breastfeeding in Saudi Arabia, it was found that almost 50% of primary health care working mothers in Al-Ahsa exclusively breastfed their infants [13]. In another research, the prevalence of exclusive breastfeeding for 6 months was 27.6% [14]. These findings revealed that exclusive breastfeeding rates in the country significantly fell short of the global targets. Furthermore, a range of studies conducted within the Saudi Arabian context have identified an array of factors that influence exclusive breastfeeding practices. Early return to work, deficient breastfeeding work support, insufficient breast milk and lack of time were the major barriers to exclusive breastfeeding [13]. These studies have examined socio-demographic, cultural, and economic determinants that shape the infant feeding behaviors of Saudi mothers [12,13,14,15,16,17]. For instance, previously published studies indicate that maternal education and occupation were associated with exclusive breastfeeding in Saudi Arabia, with better-educated and non-working mothers being more likely to engage in exclusive breastfeeding [14,17].

### 1.2. Socio-Demographic Factors

Maternal age, education, employment status, and family income are important socio-demographic factors that have consistently surfaced as determinants of exclusive breastfeeding in the literature. These factors are indicative of the complex interplay of individual, family, and societal dynamics that impact the infant feeding choices made by mothers. 

Maternal age as a critical socio-demographic factor has been examined in various studies investigating exclusive breastfeeding practices. Younger mothers often face distinct challenges related to exclusive breastfeeding, including limited access to information, lower socio-economic status, and less stable family structures [14,16,17]. In contrast, older mothers may have greater access to resources, better decision-making capabilities, and social support networks that facilitate exclusive breastfeeding. 

Maternal education is another socio-demographic factor that influences exclusive breastfeeding. In Saudi, mothers with higher levels of education tend to be better informed about the health benefits of breastfeeding, possess higher health literacy, and have greater access to informational resources, all of which contribute to their likelihood of exclusively breastfeeding their infants [15].

Employment status is also a determinant of exclusive breastfeeding. Employed mothers often face time constraints, challenges in securing appropriate breastfeeding breaks and facilities at the workplace, and the need to balance work and family responsibilities [17]. Such factors can hinder their ability to breastfeed exclusively. In contrast, non-working mothers typically have more flexible schedules and greater control over their daily routines, enabling them to practice exclusive breastfeeding more readily. 

Family income as a socio-economic factor plays a vital role in influencing exclusive breastfeeding practices. Mothers from higher-income families often have better access to healthcare services, nutritional resources, and support systems, all of which can facilitate exclusive breastfeeding [13,15,16]. Conversely, mothers with lower family incomes may face economic constraints that limit their ability to practice exclusive breastfeeding [12,16,17]. While direct evidence of this association in Saudi Arabia is limited, international studies have consistently demonstrated the impact of income disparities on exclusive breastfeeding practices.

### 1.3. Workplace Policies and Barriers

The compatibility of exclusive breastfeeding with mothers’ employment is a crucial dimension of this infant feeding practice, particularly in urban settings. Workplace-related factors, including maternity leave policies and the feasibility of expressing and storing breast milk at work, can significantly impact mothers’ ability to engage in exclusive breastfeeding [17]. Workplace policies encompass various elements that are essential for supporting exclusive breastfeeding among employed mothers. A crucial component of these policies is maternity leave, which determines the duration of time that mothers can take off work after giving birth [18,19,20,21,22,23]. Adequate maternity leave allows for mothers to exclusively breastfeed during the crucial first six months of an infant’s life. Furthermore, policies that facilitate breast milk expression and storage at the workplace are essential. Employed mothers often rely on expressing and storing breast milk to ensure that their infants continue to receive breast milk while they are at work [20,21]. Workplace support, such as designated lactation rooms and flexible break times, is crucial for this purpose. Studies have highlighted that when mothers have access to more extended maternity leave and the facilities needed to express and store breast milk at work, they are more likely to engage in exclusive breastfeeding [18,20,21,23].

Balancing employment and exclusive breastfeeding can be particularly challenging in urban settings, such as Makkah, Saudi Arabia. Urban areas often have higher workforce participation rates and different employment dynamics compared to rural regions. In urban settings, mothers may have longer commutes, encounter more rigid workplace cultures, and face additional stressors. These urban-specific challenges can further underscore the significance of supportive workplace policies and facilities for exclusive breastfeeding.

### 1.4. Significance of the Study

Despite these valuable insights into the determinants of exclusive breastfeeding in Saudi Arabia, there needs to be more research that specifically fits into the unique urban context of Makkah. The city of Makkah stands as a distinctive metropolitan area within Saudi Arabia, characterized by its socio-cultural dynamics and geographical uniqueness, thereby establishing the rationale for this research. Understanding the determinants and barriers affecting exclusive breastfeeding in Makkah becomes important as it offers insights that are useful in shaping and refining public health strategies, healthcare provider approaches, policy development, and subsequent research endeavors, all of which are designed to enhance the well-being of the infant population and ensure the optimal development of children in the region. The uniqueness of Makkah, marked by its cultural significance and the influx of millions of pilgrims and tourists annually for Hajj and Umrah, provides an intriguing and dynamic context for the exploration of breastfeeding behaviors, making this research even more essential and timely.

At the Maternity and Children’s Hospital in Mecca, medical care is provided for women and children after childbirth in a dedicated postnatal ward, with shared rooms with another patient, and the child remains with the mother to enable breastfeeding. The hospital has large postnatal wards that are not family-friendly and do not allow partners to stay overnight unless the mothers are having a Cesarean section (C-section). Moreover, mothers are evaluated for comfort and confidence in breastfeeding to overcome challenges, including pain that comes with breastfeeding. 

### 1.5. Research Objectives

This study aims to investigate the various factors influencing exclusive breastfeeding practices in Makkah, Saudi Arabia. The objectives of this research are as follows:

I. To identify the socio-demographic factors associated with exclusive breastfeeding in Makkah.
II. To explore the influence of healthcare professionals, family support, and workplace policies on exclusive breastfeeding practices.
III. To examine the barriers and challenges faced by mothers in Makkah in their pursuit of exclusive breastfeeding.

## 2. Materials and Methods

### 2.1. Research Design

This study employs a cross-sectional research design to assess the factors affecting breastfeeding practices among mothers attending the Maternity and Childhood Hospital in Makkah Al-Mukarramah, Saudi Arabia. This renowned government hospital, affiliated with the Saudi Ministry of Health, is dedicated to maternal and child healthcare, offering a wide range of services. The hospital features both internal and external clinics, medical insurance coverage, comprehensive medical departments, a distinguished medical staff, modern medical equipment, and round-the-clock service availability. 

### 2.2. Study Sample

The study sample consisted of 340 infants and their mothers. Data were gathered through a questionnaire administered to mothers during their visits to the Well-Baby Clinics at the Maternity and Child Hospital in Makkah, Saudi Arabia. The sample selection criteria included infants aged between 6 and 24 months, and mothers attending the hospital for healthcare services. The data were collected over three months from July to October 2023.

### 2.3. Study Tool

The primary data collection instrument employed in this study was a structured questionnaire. The questionnaire was adapted from a previously validated instrument [12] developed by Radwan and Hadia (2013). The questionnaire is divided into several main sections:

Part One: Personal Information: This section collects demographic and functional data about the study sample, including age, educational level, and mother’s occupation.

Part Two: Health Information of Mother and Child: This section gathers information regarding the number of babies, weight, and gender of the child, and the presence of any maternal diseases.

Part Three: This part focuses on aspects related to breastfeeding practices, including the timing of initiation of breastfeeding, the duration of breastfeeding, the number of daily breastfeeding sessions, and the type of feeding (breastfeeding or formula milk).

Part Four: This section seeks to capture data on breastfeeding and food habits in the first two years of the child’s life.

### 2.4. Ethical Consideration

Ethical approval for this study was obtained from the Saudi Ministry of Health Institution Review Board Committee (H-02-K-076-0723-977). Informed consent was requested to be read by participants who satisfied the inclusion requirements. Participants were informed that this study on the factors influencing exclusive breastfeeding practices is voluntary and that they are free to refuse to answer any questions or to leave the study at any moment. 

### 2.5. Data Analysis

Data were analyzed using the SPSS statistical software Version (27). In this cross-sectional study, descriptive statistics were utilized to summarize and present the characteristics of the study sample and the variables of interest. Inferential statistics were employed to examine relationships, associations, and differences among variables. For instance, ANOVA allowed for the exploration of variations in breastfeeding practices among mothers with different characteristics, such as the number of children or the presence of maternal diseases. Multiple regression was performed to assess the simultaneous effect of several predictor variables, such as maternal age, educational qualification, and the presence of maternal diseases, on the duration and frequency of breastfeeding.

## 3. Results

A total of 340 valid questionnaires from study participants were included in this analysis. Overall, 100% of the participants addressed each data variable in the survey tool to determine factors influencing the exclusive breastfeeding in Saudi Arabia. 

### 3.1. Part 1: Demographic Data of the Study Participants

The demographic profiles of the study participants presented in Table 1 revealed a diverse sample of mothers with infants attending the Maternity and Childhood Hospital in Makkah Al-Mukarramah, Saudi Arabia. Among the 340 individuals included in the study, 325 were Saudi nationals, whereas 15 were categorized as “non-Saudi” from the nationality perspective. Most of the sample reported to be from families of five or fewer people and earning income between SAR 5000 and 10,000. Concerning educational levels, most mothers (83.8%) possessed a university education, reflecting a highly educated sample. In addition, the results indicated that 54.5% of mothers were aged less than 40 years compared to 45.5% of them aged more than 40 years. Similarly, the fathers’ educational levels exhibited a notable emphasis on university education (85.6%). 

### 3.2. Part 2: Health Information about the Mother and Child

The majority of participants had a natural birth (72.1%), gave birth to a male baby (53.8%), had a baby whose birth was classified as the ninth birth (93.8%), had a baby with a birth weight between 2.6 and 3.0 kg (56.2%) and were experiencing their second birth (37.1%). Furthermore, a significant portion of mothers did not suffer from health diseases (96.8%), and their babies did not experience post-birth illnesses (97.4%). In terms of pregnancy control methods, non-hormonal methods were the most commonly used (40.6%). While the majority of mothers were non-smokers (92.1%), more than half of the families had at least one family member who smoked (50.3%). Most babies were in the mother’s room after birth (77.6%).

### 3.3. Part 3: Postpartum Information

Postpartum information within the study revealed several key factors related to breastfeeding practices. Most mothers (74.4%) initiated their first breastfeeding within an hour after birth, emphasizing the significance of timely breastfeeding initiation in this population. Here, it becomes clear to us that most mothers who initiate breastfeeding in the first hours after childbirth prefer to have their child stay with them in the same room. Additionally, the predominant duration of exclusive breastfeeding without formula was 0–6 months (88.5%), aligning with the recommended exclusive breastfeeding period. A notable proportion of mothers (56.5%) reported using a pacifier to soothe their infants, indicating a common practice within the study population. Most mothers (91.5%) reported breastfeeding according to their child’s desire rather than adhering to a regular appointment schedule, underlining the importance of responsive and demand-based breastfeeding practices. Furthermore, in terms of breastfeeding termination, formula milk was the most common method chosen (62.9%), indicating a shift away from exclusive breastfeeding, while a substantial number of mothers (33.2%) reported that breastfeeding was the most likely mode of feeding for their infants at this stage.

### 3.4. Part 4: Information about Breastfeeding and Dietary Habits during the Breastfeeding Period

The data regarding the provision of information about breastfeeding before giving birth indicated that about half of the mothers in the study population received such information. Furthermore, when considering the health status of the babies after feeding, most of the infants did not experience health problems following their feeding.

#### Multiple Regression Analysis

The first regression analysis aimed to identify the factors influencing the duration of exclusive breastfeeding (without the introduction of formula) among mothers attending the Maternity and Childhood Hospital in Makkah, Saudi Arabia. The findings revealed that among the examined factors, mother’s age exhibited a noteworthy positive effect on the duration of exclusive breastfeeding. This suggests that older mothers tend to engage in exclusive breastfeeding for a longer duration. However, it is crucial to note that this effect, although moderate, did not reach statistical significance at the conventional alpha level of 0.05, with a *p*-value of 0.065. In contrast, other variables such as nationality, family size, family income level, mother’s educational level, father’s education level, and mother’s employment status did not demonstrate statistically significant effects on the duration of exclusive breastfeeding within this study population (see Table 2).

The second regression analysis was conducted to explore the determinants of exclusive breastfeeding duration. The method of childbirth, as represented by the variable “Type of birth”, emerged as a statistically significant factor influencing the duration of exclusive breastfeeding. Mothers who had a Cesarean section (C-section) delivery tended to engage in shorter periods of exclusive breastfeeding compared to those who had natural deliveries. Although this finding is statistically significant (*p*-value = 0.043), it also highlights the need for further research to explore the specific factors and experiences that contribute to this association. In contrast, other variables such as the nature of the current birth (first child or subsequent child), the gender of the baby, birth order, baby weight at birth, the presence of maternal health diseases, and infant diseases after birth did not demonstrate statistically significant effects on the duration of exclusive breastfeeding in this study population. These non-significant findings suggest that factors beyond those explored in this analysis may be influential, and a more extensive investigation may be necessary to uncover the full spectrum of determinants affecting exclusive breastfeeding practices (see Table 3).

The last regression analysis offers valuable insights into the determinants of exclusive breastfeeding duration, shedding light on the factors that influence this critical aspect of infant nutrition. Notably, the analysis revealed that the timing of the first breastfeed and whether the mother received information about breastfeeding before giving birth significantly impact the duration of exclusive breastfeeding. Mothers who initiated breastfeeding early and did not receive information before childbirth tended to engage in longer periods of exclusive breastfeeding. While several other factors, including maternal and family smoking, pregnancy control methods, the baby’s location after birth, and the use of pacifiers, did not exhibit significant effects in this study population, the identified determinants underscore the multifaceted nature of maternal practices in infant nutrition, emphasizing the need for comprehensive support strategies to promote exclusive breastfeeding.

## 4. Discussion

### 4.1. Early Initiation and Breastfeeding Information

One of the findings of this study is the significant influence of the timing of the first breastfeed and the receipt of information about breastfeeding before childbirth on the duration of exclusive breastfeeding. Mothers who initiated breastfeeding early and those who did not receive pre-birth breastfeeding information tended to engage in longer periods of exclusive breastfeeding. These findings highlight the pivotal role of healthcare education and support in promoting optimal infant feeding practices [24,25,26,27,28]. Encouraging early breastfeeding initiation and ensuring mothers are equipped with essential information before childbirth are critical strategies for fostering exclusive breastfeeding.

The findings underscore the need for healthcare facilities and policymakers to prioritize the integration of breastfeeding education and support into maternal and child health programs. Ensuring that mothers receive comprehensive information and assistance related to breastfeeding before giving birth can have a profound impact on exclusive breastfeeding practices [27]. The evidence from this study further reinforces the call for healthcare professionals to engage in proactive counseling and education about breastfeeding, emphasizing the advantages of early initiation and exclusive breastfeeding. As such, healthcare providers and policymakers must recognize the pivotal role they play in facilitating maternal and child health by enabling mothers to make informed and empowered decisions regarding infant nutrition, which, in turn, leads to longer durations of exclusive breastfeeding and the many associated health benefits for infants.

In 2023, the Maternity and Child Hospital in Makkah passed and succeeded in implementing the Baby-Friendly Hospital Initiative (BFHI). According to WHO and UNICEF standards, the hospital provides all necessary means and medical care by all its staff to encourage breastfeeding. Through the Saudi Ministry of Health, health practitioners were trained on how to support and encourage mothers to breastfeed. 

### 4.2. Maternal and Family Smoking

While maternal smoking and the presence of family members who smoke appeared to have positive effects on the duration of exclusive breastfeeding, these effects were not statistically significant. The non-significant findings suggest that while these factors may have some influence, their impact is not strong enough to reach statistical significance in this study population. Nevertheless, it is important to consider the potential health risks associated with smoking, and mothers should be advised to quit or avoid smoking during pregnancy and lactation to ensure the well-being of both mother and child [29,30,31,32].

Maternal and family smoking should be addressed not only in the context of breastfeeding but also as a broader public health concern. Policies and programs aimed at reducing maternal smoking rates and preventing exposure to secondhand smoke are essential to protect the health of infants and children [31,32]. Healthcare professionals play a pivotal role in providing guidance and support to mothers who smoke, emphasizing the advantages of quitting or avoiding smoking during pregnancy and lactation [29]. Furthermore, it is essential to create smoke-free environments for children, as this not only benefits their immediate health but also contributes to the promotion of exclusive breastfeeding, a cornerstone of infant health and development.

### 4.3. Cesarean Section and Exclusive Breastfeeding

The study also identified a statistically significant factor affecting exclusive breastfeeding duration, which is the type of childbirth method. Mothers who underwent Cesarean section (C-section) deliveries tended to engage in shorter durations of exclusive breastfeeding compared to those who had vaginal deliveries. This finding is in line with previous research, highlighting the potential challenges or barriers faced by mothers who undergo C-sections, which may hinder their ability to sustain exclusive breastfeeding [33,34,35].

It is imperative to provide additional support and guidance to mothers who have C-section deliveries to help them overcome these challenges and promote extended exclusive breastfeeding practices [36]. The findings emphasize the importance of maternity care practices that promote skin-to-skin contact and breastfeeding immediately after C-section deliveries. Healthcare facilities should adopt protocols that prioritize breastfeeding support for mothers who have had C-sections, ensuring that they receive appropriate guidance and assistance to overcome potential barriers [36,37].

The promotion of exclusive breastfeeding is essential not only for the immediate health benefits for infants but also for the long-term health outcomes associated with breastfeeding. By addressing the challenges faced by mothers who undergo C-sections and providing them with the necessary support, healthcare professionals and policymakers can contribute to the promotion of extended exclusive breastfeeding practices and, in turn, enhance the health and well-being of both mothers and infants.

## 5. Conclusions

### 5.1. Recap of Key Findings

The study provided valuable insights into the determinants of exclusive breastfeeding practices among mothers attending the Maternity and Childhood Hospital in Makkah, Saudi Arabia. Notable findings include the positive impact of early breastfeeding initiation and the absence of pre-birth breastfeeding information on the duration of exclusive breastfeeding. These findings underscore the crucial role of healthcare education and timely support in promoting optimal infant feeding practices. Moreover, the study identified the significant influence of the method of childbirth, with Cesarean section deliveries associated with shorter durations of exclusive breastfeeding. These findings emphasize the importance of tailored support for mothers who underwent C-sections to overcome potential barriers to extended exclusive breastfeeding.

### 5.2. Implications for Practice and Policy

The findings of this study have several practical and policy implications. Healthcare professionals should prioritize early breastfeeding initiation and provide comprehensive breastfeeding information to expectant mothers. This could be achieved through prenatal education programs and postnatal support in healthcare facilities. Additionally, special attention should be given to mothers who have C-section deliveries, as they may face unique challenges in maintaining exclusive breastfeeding. Healthcare policies should ensure that adequate support is available to address these challenges, including strategies to promote skin-to-skin contact and breastfeeding immediately after C-section deliveries.

### 5.3. Recommendations for Future Research

While this study provides valuable insights, there are opportunities for future research to expand upon these findings. Research with a larger and more diverse sample size could confirm the significance of factors that did not reach statistical significance in this study. Longitudinal research designs can help establish causal relationships between determinants and exclusive breastfeeding duration, providing a more comprehensive understanding of the dynamics involved. Additionally, qualitative research approaches may offer a deeper exploration of the experiences and perceptions of mothers, healthcare providers, and family members regarding exclusive breastfeeding practices.

## Figures and Tables

**Table 1:** Demographic profile of participants (N = 340).

Nationality			
	Frequency	Percent	Valid Percent
Saudi	325	95.6	95.6
Non-Saudi	15	4.4	4.4
Total	340	100.0	100.0
**Family size**			
	Frequency	Percent	Valid Percent
5 or less	282	82.9	82.9
6 or more	58	17.1	17.1
Total	340	100.0	100.0
**Mother’s age**			
	Frequency	Percent	Valid Percent
20–24 years old	39	11.5	11.5
25–29 years old	116	34.1	34.1
30–34 years old	106	31.2	31.2
35 years old and above	79	23.2	23.2
Total	340	100.0	100.0
**Family income level**			
	Frequency	Percent	Valid Percent
Less than SAR 5000	19	5.6	5.6
SAR 5000–10,000	176	51.8	51.8
More than SAR 10,000	145	42.6	42.6
Total	340	100.0	100.0
**Mother’s educational level**			
	Frequency	Percent	Valid Percent
High education	20	5.9	5.9
Middle	35	10.3	10.3
University	285	83.8	83.8
Total	340	100.0	100.0
**Father’s education level**			
	Frequency	Percent	Valid Percent
High education	25	7.4	7.4
Middle	24	7.1	7.1
University	291	85.6	85.6
Total	340	100.0	100.0
**Is the mother working?**			
	Frequency	Percent	Valid Percent
Yes	120	35.3	35.3
No	220	64.7	64.7
Total	340	100.0	100.0
**Did the mother receive information about breastfeeding before giving birth?**			
	Frequency	Percent	Valid Percent
Yes	183	53.8	53.8
No	157	42.2	42.2
Total	340	100.0	100.0

**Table 2:** Factors influencing the duration of exclusive breastfeeding.

Model	B	Std. Error	Beta	t	*p*
(Constant)	0.731	0.539		1.355	0.176
Nationality	0.306	0.220	0.079	1.391	0.165
Family size	−0.066	0.143	−0.031	−0.463	0.643
Mother’s age	0.102	0.055	0.122	1.852	0.065
Family income level	−0.039	0.086	−0.029	−0.457	0.648
Mother’s educational level	0.076	0.087	0.052	0.880	0.379
Father’s education level	−0.092	0.082	−0.065	−1.122	0.263
Is the mother working?	0.098	0.100	0.059	0.981	0.327
Dependent Variable: How long did you breastfeed without formula?					

**Table 3:** Determinants of exclusive breastfeeding duration.

Model	B	Std. Error	Beta	t	*p*
(Constant)	−0.047	0.842		−0.056	0.956
Is the current birth a first, second, third, or more?	0.053	0.044	0.065	1.198	0.232
Type of birth	−0.199	0.098	−0.112	−2.031	0.043
The sex of the baby	0.141	0.087	0.088	1.631	0.104
the birth of the child	0.117	0.190	0.035	0.616	0.538
Baby weight at birth	0.117	0.069	0.098	1.705	0.089
Does the mother suffer from health diseases?	0.336	0.243	0.074	1.380	0.169
Did the child suffer from diseases after birth?	0.056	0.269	0.011	0.209	0.835
(Constant)	1.084	0.396		2.737	0.007
Type of pregnancy control method	−0.034	0.055	−0.033	−0.617	0.538
Is the mother a smoker?	0.283	0.159	0.096	1.782	0.076
Is anyone in the family a smoker?	0.135	0.086	0.084	1.567	0.118
Where is the baby after birth?	0.046	0.128	0.024	0.361	0.719
What time does the first breastfeed start?	−0.304	0.121	−0.166	−2.515	0.012
Do you use a pacifier to silence the baby?	0.083	0.088	0.052	0.942	0.347
Did the mother receive information about breastfeeding before giving birth?	−0.199	0.087	−0.124	−2.278	0.023
Dependent Variable: How long did you breastfeed without formula?					

## Data Availability

The datasets analyzed during the current study are available from the author on reasonable request.
